# *Magnaporthe oryzae* effector AvrPik-D targets a transcription factor WG7 to suppress rice immunity

**DOI:** 10.1186/s12284-024-00693-0

**Published:** 2024-02-13

**Authors:** Tao Yang, Linlin Song, Jinxian Hu, Luao Qiao, Qing Yu, Zonghua Wang, Xiaofeng Chen, Guo-dong Lu

**Affiliations:** 1 State Key Laboratory of Ecological Pest Control for Fujian and Taiwan Crops, College of Plant Protection, Fujian Agriculture and Forestry University, Fuzhou, 35002 China; 2 Fujian Universities Engineering Research Center of Marine Biology and Drugs, Fuzhou Institute of Oceanography, College of Geography and Oceanography, Minjiang University, Fuzhou, 350108 China; 3 Ministerial and Provincial Joint Innovation Centre for Safety Production of Cross-Strait Crops, Minjiang University, Fuzhou, 350108 China

**Keywords:** *Oryza sativa*, *Magnaporthe oryzae*, Avirulence factor, Transcription factor, Rice immunity

## Abstract

Rice blast, caused by the fungal pathogen *Magnaporthe oryzae*, is one of the most devastating diseases for rice crops, significantly affecting crop yield and quality. During the infection process, *M. oryzae* secretes effector proteins that help in hijacking the host's immune responses to establish infection. However, little is known about the interaction between the effector protein AvrPik-D and the host protein Pikh, and how AvrPik-D increases disease severity to promote infection. In this study, we show that the *M. oryzae* effector AvrPik-D interacts with the zinc finger-type transcription factor WG7 in the nucleus and promotes its transcriptional activity. Genetic removal (knockout) of the gene *WG7* in transgenic rice enhances resistance to *M. oryzae* and also results in an increased burst of reactive oxygen species after treatments with chitin. In addition, the hormone level of SA and JA, is increased and decreased respectively in *WG7* KO plants, indicating that WG7 may negatively mediate resistance through salicylic acid pathway. Conversely, *WG7* overexpression lines reduce resistance to *M. oryzae.* However, WG7 is not required for the Pikh-mediated resistance against rice blast. In conclusion, our results revealed that the *M. oryzae* effector AvrPik-D targets and promotes transcriptional activity of WG7 to suppress rice innate immunity to facilitate infection.

**Supplementary Information:**

The online version contains supplementary material available at 10.1186/s12284-024-00693-0.

## Introduction

Rice (*Oryza sativa* L.), the main food crop that feeds more than half of the world's population, is under serious threat from diseases caused by various pathogens (Zeng et al. 2017). To combat pathogen infections, plants evolved two branches of the immune system: pathogen-associated molecular patterns (PAMPs)-triggered immunity (PTI) and effector-triggered immunity (ETI) (Jones and Dangl 2006; Han et al. 2021). PTI and ETI induce immune responses such as the burst of reactive oxygen species (ROS), elevation of cytosolic Ca^2+^, and activation of kinase cascades, further responses include pathogenesis-related (*PR*) gene expression and deposition of phenolic compounds (Boller and Felix 2009; Park et al. 2012; Bigeard et al. 2015; Gao et al. 2021).

*Magnaporthe oryzae*, an airborne pathogenic fungus that causes rice blast, is highly adaptable and can infect rice at all stages of development (Lee 1994). The adaptability of *M. oryzae* mainly results from mutations in the pathogen, leading to the instability of avirulence (*AVR*) genes, thereby overcoming the major *R* gene-mediated resistance (Dai et al. 2010). Therefore, it is essential to continue identifying and cloning new effective genes and their alleles for *R* gene-mediated resistance (Navadagi B. Devanna et al. 2014). Most intracellular R protein in plants are nucleotide-binding/leucine-rich repeat (NLR) receptors that can be subdivided into Toll/Interleukin-1 receptor/resistor (TIR)-NLR (TNL) and coil (CC)-NLR (CNL) based on their N-terminal domains (Tamborski and Krasileva 2020). To date, more than 100 blast *R* genes have been mapped in the rice genome, and 38 of them have been successfully cloned (Wang et al. 2017; Liu et al. 2023). Among the cloned *R* genes, 9 corresponding *Avr* genes of *M. oryzae* have also been cloned (Wang et al. 2017). In these avirulence genes, the encoded proteins of *AvrPita*, *Avr1CO39*, *AvrPia*, *AvrPik*, and *AvrPi54* directly interact with cognate R proteins to trigger the R protein-mediated immunity response (Yulin Jia et al. 2000; Kanzaki et al. 2012; Cesari et al. 2013; Navadagi B. Devanna et al. 2014).

In contrast, several *Avr* proteins, including AvrPiz-t, AvrPii, AvrPib, and AvrPi9, interact indirectly with congener R proteins to initiate R protein-mediated disease resistance (Park et al. 2012; Fujisaki et al. 2015; Wu et al. 2015; Xie et al. 2022). In nature, *Avr* protein is a virulence factor to host plant without corresponding resistance gene. However, the virulence function of only a few *Avr* proteins of *M. oryzae* have been characterized. For instance, AvrPita manipulates host immunity by interacting with a cytochrome c oxidase (COX) assembly protein OsCOX11, thereby inhibiting ROS accumulation and rice immunity (Han et al. 2021). AvrPiz-t inhibits the E3 ligase activity of the RING-type ubiquitin E3 ligases APIP6 and APIP10, and promotes their degradation, thereby manipulating host immunity (Park et al. 2012, 2016). Inside the host cell, AvrPiz-t targets the bZIP-type transcription factor APIP5 to weaken APIP5-triggered cell death and promote tissue necrosis (Wang et al. 2016). APIP5 directly binds to the promoters of *OsWAK5* and *CYP72A1* genes to inhibit their expression, consequently limiting lignin accumulation, ROS production, and defense compound accumulation (Zhang et al. 2022). AvrPi9 targets OsRGLG5 and affected the stability of OsRGLG5 to suppress basal resistance against *M. oryzae*, whereas OsRGLG5 ubiquitinates and degrades AvrPi9 (Liu et al. 2023). AvrPi9 also interacts with ANIP1 to modulate rice immunity in distinct ways in the presence or absence of the corresponding resistance protein (Shi et al. 2023). AvrPii targets the rice NADP-malic enzyme to regulate ROS accumulation and suppress host basal resistance (Singh et al. 2016). These evidence shows that *Avr* proteins target diverse host factors to manipulate host immunity.

*Pik-h*, an allele of *Pik*, confers resistance against the rice blast pathogen *M. oryzae* with a pair of CC-type NBS-LRR, *Pikh-1* and *Pikh-2*, which are genetically linked in head-to-head orientation, and Pikh-1 CC (coiled coil) domain interacts directly with both AvrPik-h (also called AvrPik-D) and Pikh-2 (Zhai et al. 2014). Both the Pik-1/Pik-2 pairs and AvrPik effectors are represented as part of an allelic series in rice and *M. oryzae* populations (Xiao et al. 2023). At least nine *Pik* alleles, including Pik*, *Pikm*, *Pikp*, *Pikh*, *Pi1*, *Pi7*, *Pik*, *Pike* and *Piks*, have been identified (Campbell et al. 2004; Ashikawa et al. 2008, 2011; Yuan et al. 2011; Zhai et al. 2011, 2014; Hua et al. 2012; Kanzaki et al. 2012; Chen et al. 2015; Xiao et al. 2023). Six variants (alleles) of *AvrPik* (A–F) each differ by 1–5 amino substitutions (Longya et al. 2019; Xiao et al. 2023). At present, the molecular mechanism of recognizing *AvrPik* variants by *Pik* alleles has been extensively investigated. While, little is known about the pathogenesis of AvrPik in rice. It was reported that AvrPik bound the rice HMA proteins OsHIPP19 and OsHIPP20 to stabilize their proteins to suppresses host defenses, knockout of *OsHIPP20* causes enhanced disease resistance to the blast pathogen (Oikawa et al. 2020; Maidment et al. 2021). Our previous studies also have shown that AvrPik-D is secreted into plant cells by the BIC structure to inhibit basal resistance to *M.oryzae* (date not shown). However, the mechanisms by which AvrPik-D manipulates plant immunity remain unclear.

In this study, we revealed that AvrPik-D primarily targets the host nucleus where it interacts with a transcription factor protein, WG7. This interaction facilitates the enhancement of WG7's transcriptional activity, leading to the suppression of the ROS burst, and thus inhibiting innate immunity in the Pikh-lacking cultivar NPB. Moreover, we observed that this interaction is not necessary for Pikh-mediated recognition of AvrPik-D in the Pikh background.

## Results

### AvrPik-D interacts with the CW-type zinc figner transcription factor WG7

To elucidate the molecular mechanism underlying the suppression of host PTI mediated by AvrPik-D, we performed a yeast two-hybrid (Y2H) assay using AvrPik-D^22−113^ (which lacks the N-terminal signal peptide, ΔSP) as bait to identify potential host targets of AvrPik-D. By screening a blast fungus infection rice cDNA library, we identified a candidate cysteine-tryptophan (CW)-type Zinc Finger protein which we named AKIP695 (AvrPik-D interacting protein, *LOC_Os07g47360*). In rice, the *AKIP695* gene consists of a 4800 bp open reading frame that encodes a protein of 1600 amino acids (Additional file 1: Fig. S1A). The AKIP695 regultes rice grain width therefore named as WG7 in the previous studies. It is involved in histone recognition (Zhang et al. 2017) and functions as a transcriptional activator for regulating gene expression (Huang et al. 2020). Therefore, we rename AKIP695 as WG7.

**Fig. 1:**
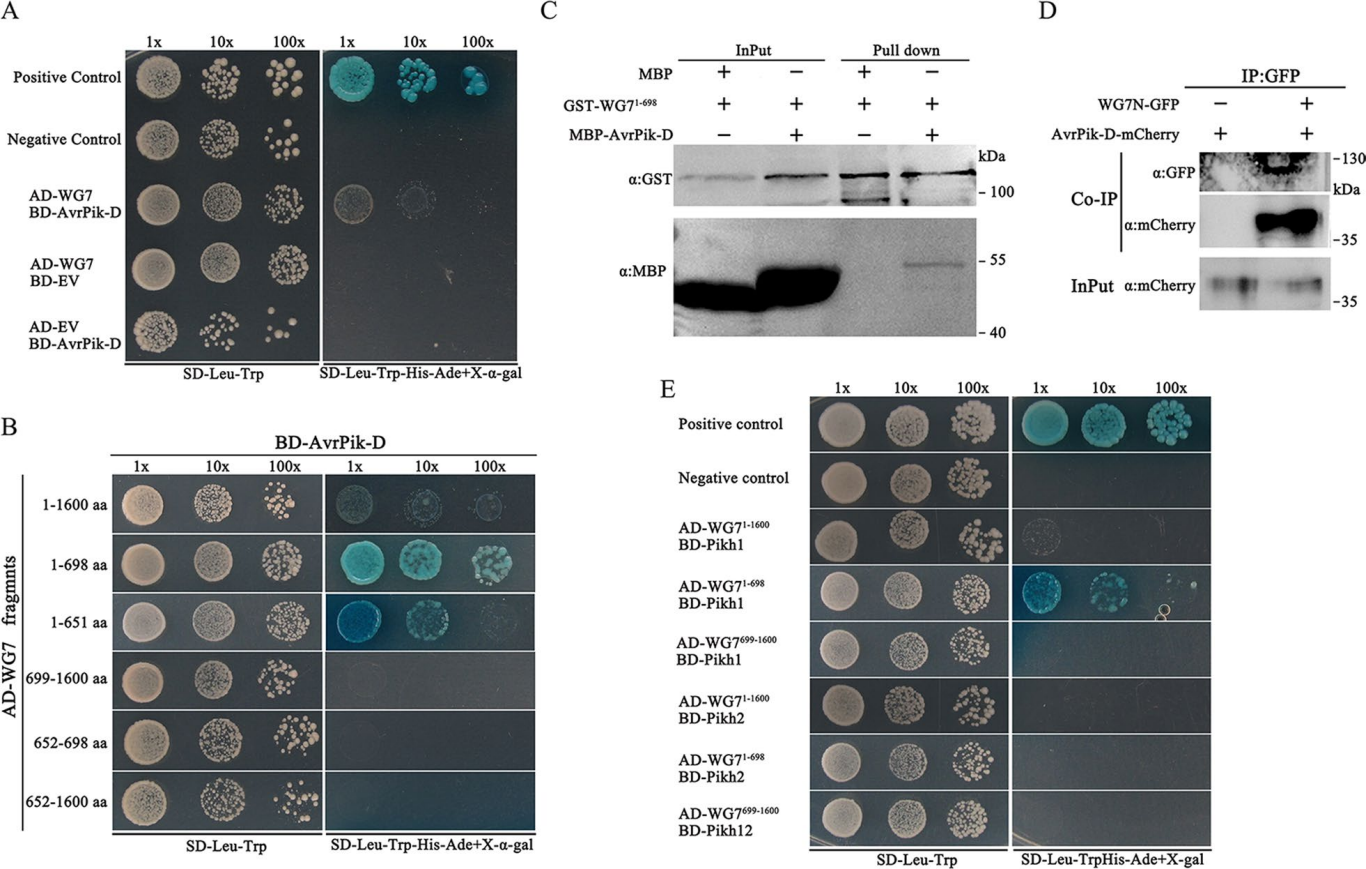
AvrPik-D interacts with WG7. **A** Analysis of protein–protein interactions between BD:AvrPik-D (ΔSP) and AD:WG7 via yeast-two-hybrid (Y2H) assay. Yeast cells harboring the indicated bait and prey plasmids were diluted 1 × , 10 × , and 100 × , and then spotted onto selective media SD-Leu-Trp and SD-Leu-Trp-His-Ade with X-α-gal. The positive and negative controls were pGADT7-T + pGBKT7-53 and pGADT7-T + pGBKT7-lam. SD (-Leu-Trp) indicates selective medium lacking leucine and tryptophan. SD (-Leu-Trp-His-Ade) indicates selective medium lacking leucine, tryptophan, adenine and histidine. **B** Deletion assays of WG7 to test interactions with AvrPik-D. A series of truncated WG7 proteins were tested for interaction with AvrPik-D via Y2H. **C** In vitro pull-down assays were conducted with fusion proteins (GST:WG7^1−698^ and MBP:AvrPik-D) using GST beads and detected by immunoblot analysis with anti-GST and anti-MBP antibodies. **D** Co-immunoprecipitation (Co-IP) assay of WG7N-GFP and AvrPik-D-mCherry in *N. benthamiana*. The Co-IP assay (IP) was carried out with anti-GFP beads, and the proteins were analyzed by western blot with anti-GFP and anti-mCherry antibodies. **E** Y2H assay to detect the interaction among WG7^1−1600^, WG7^1−698^, WG7^699−1600^, Pikh1 and Pikh2.

We cloned the full-length coding sequence of *WG7* CDS and confirmed the interaction between AvrPik-D and WG7 in Y2H assay (Fig. 1A). Sequence analysis revealed that the WG7 has a CW domain (aa 652–698). To define the region of the WG7 protein interacting with AvrPik-D, we generated a series of truncated WG7 fragments for Y2H assays (Additional file 1: Fig S1A). The results showed that the WG7^1−698^ fragment encompasses a sufficient region for interaction with AvrPik-D. Interestingly, the WG7^1−651^ fragment, which does not contain the CW domain, was also found to be critical for the interaction. Notably, a strong interaction was observed if the WG7^1−698^ fragment includes the CW domain, indicating the CW domain's involvement in their interaction (Fig. 1B). We then performed a Glutathione S-transferase (GST) pull-down assay using the GST-WG7^1−698^ fragment (N-terminal) and MBP-AvrPik-D (ΔSP). Immunoblot analysis showed that the GST-fused WG7^1−698^ proteins bound to MBP-AvrPik-D, but not to MBP alone (Fig. 1C), indicating that the N-terminal of WG7 interacts with AvrPik-D in vitro. To confirm that the interaction also occurs in vivo, we used the co-immunoprecipitation (CoIP) method. The plasmids of AvrPik-D (ΔSP) fused with the C terminus mCherry tag (AvrPik-D-mCherry) and the N-terminal WG7^1−698^ fused with the C terminus GFP tag (WG7N-GFP), were either co-expressed or separately expressed in *Nicotiana benthamiana* leaves using the agroinfiltration method. When the WG7N-GFP fusion protein was immunoprecipitated from the plant extract using anti-GFP IgG beads, the AvrPik-D-mCherry proteins were detected in the immunocomplex of WG7N-GFP with the anti-mCherry antibody (Fig. 1D). However, no visible background signal was detected in the samples expressing only AvrPik-D-mCherry. These results indicate that AvrPik-D interacts with WG7 in vivo. Because AvrPik-h (also named AvrPik-D) is the corresponding avirulence factor of Pikh (Zhai et al. 2014; De la Concepcion et al. 2021), we tested whether WG7 interacts with Pikh. The Y2H assay showed that the N-terminal of WG7^1−698^ interacts with the full-length Pikh1 in yeast, but not Pikh2 (Fig. 1E). To further define the domain of Pikh1 protein interact with WG7^1−698^, we truncated Pikh1 to generate 4 parts (including CC, HMA, NB-ARC and LRR) for Y2H assays (Additional file 1: Fig S1B). The results showed that only the CC domain of Pikh1 interacts with WG7^1−698^ fragment, and the HMA domain of Pikh1 interacts with AvrPik-D (Additional file 1: Fig S1B). These results indicate that Pikh1 interacts with WG7 and AvrPik-D by different domain, respectively.

Six variants (alleles) of *AVR-Pik* (*AVR-PikA*, *AVR-PikB*, *AVR-PikC*, *AvrPik-D*, *AVR-PikE* and *AVR-PikF*) have been reported. These alleles differ from each other by a total of five nucleotide substitutions (Longya et al. 2019). We cloned the CDS of these *AVR-Pik* (ΔSP) alleles into the Bait (BD) vectors, then performed a Y2H assay. The result showed the interaction between AvrPik alleles and the WG7^1−698^ proteins (Additional file 1: Fig S1C). This result suggests that WG7 binds to conserved regions of AvrPik alleles. We also used the Y2H assay to test whether other *Avr* genes of *M. oryzae* interact with WG7. The results show that Avr1CO39 strongly interacts with WG7, while AvrPii exhibits a weak interaction with WG7 (Additional file 1: Fig S1D). Since Avr1CO39 and AvrPik share a similar six-stranded β-sandwich structures by known as a typical MAX (*Magnaporthe* Avrs and ToxB-like) effector family (Guillen et al. 2015; Petit-Houdenot et al. 2020), and their protein structures have been experimentally determined (Guillen et al. 2015; Maqbool et al. 2015). Although Avr1CO39 has little primary sequence similarity to the AvrPik but structurally similar when using the PyMOL server and the Protein Data Bank (Guillen et al. 2015) to perform alignment (Additional file 1: Fig S1E). This result suggests that WG7 might be bound to *Avr* proteins with similar topology structures of AvrPik, thus inhibiting plant immunity.

### WG7 is co-localized with AvrPik-D in the nucleus

**Fig. 2:**
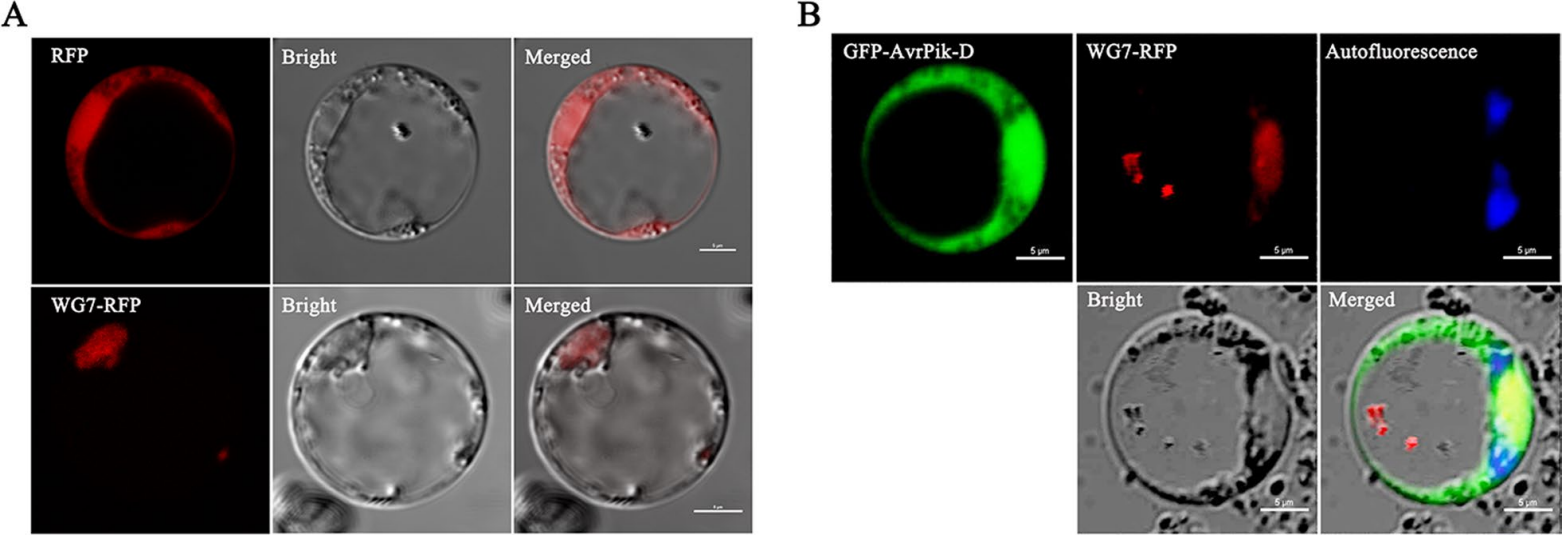
AvrPik-D and WG7 co-localize to rice nucleus. **A** WG7:RFP was expressed in rice protoplasts. RFP fluorescent signals observed in the nucleus. **B** GFP:AvrPik-D and WG7:RFP were co-expressed in rice protoplasts. GFP and RFP fluorescent signals overlapped in the nucleus. The blue fluorescent signal indicated self-luminescence. Scale bars = 5 μm

The full-length WG7 CDS was fused to RFP under the control of the Ubiquitin promoter and used to determine the subcellular localizations. Confocal microscopy showed that WG7-RFP was localized in the nucleus of rice protoplasts (Fig. 2A), and the signal of RFP and DAPI had co-localized in the nucleus (Additional file 1: Fig S2A), consistent with previous reports (Zhang et al. 2017). We further confirmed the nuclear localization of WG7 in *N. benthamiana* leaves, where WG7-GFP and histone RFP-H2B were found to be co-localized in the nucleus (Additional file 1: Fig S2C). The subcellular localization of WG7 prompted us to determine whether AvrPik-D is co-localized with WG7 in rice cells. We co-expressed WG7-RFP with GFP-AvrPik-D in rice protoplasts, the overlapped signals between WG7-RFP and GFP-AvrPik-D were observed in the nucleus (Fig. 2B), and the signal of RFP and GFP had co-localized with DAPI in the nucleus (Additional file 1: Fig S2B). To further investigate the co-localization of WG7 and AvrPik-D *in planta*, we co-infiltrated *N.benthamiana* leaves with WG7-GFP and AvrPik-D-RFP. The results were consistent with those observed in rice protoplasts (Additional file 1: Fig S2C). These results indicate that the fungal effector AvrPik-D targets the host nucleus and binds to WG7.

### WG7 negatively regulates immunity against rice blast fungus

**Fig. 3:**
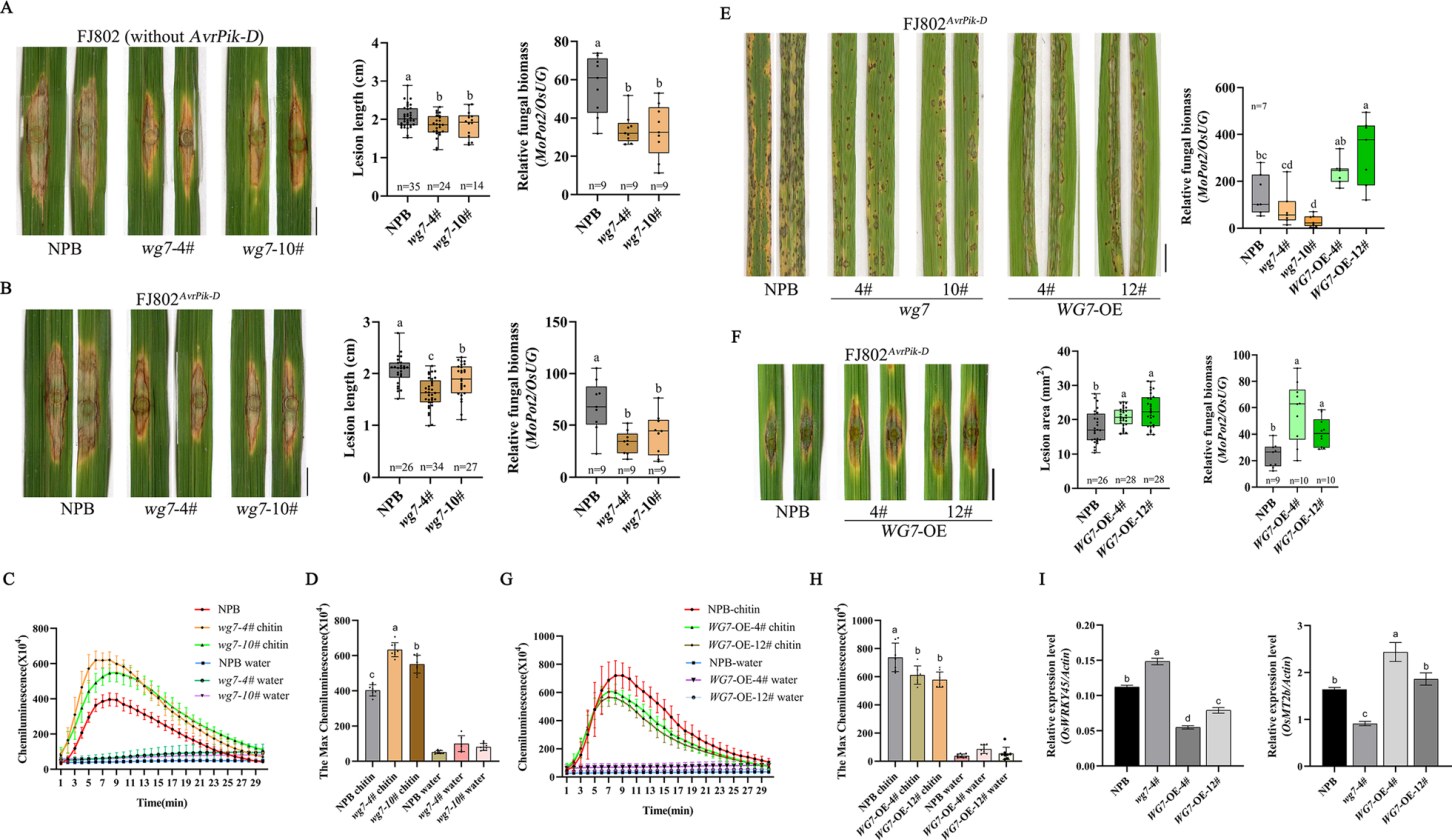
WG7 negatively regulates the basal resistance of rice against *M. oryzae*. **A**, **B** Disease symptoms of *wg7* and wild type (WT) plants after punch inoculation with *M. oryzae* strains FJ802 (without *AvrPik-D*) and FJ802^*AvrPik-D*^ (with *AvrPik-D*). The images were photographed at 10 days post inoculation (dpi). Scale bar = 0.5 cm. Lesion length was measured using ImageJ software. Data are shown as mean ± standard error (SD) (Different letter indicate *P* < 0.05, n ≥ 14). Relative fungal biomass of *M. oryzae* in inoculated leaves, as quantified by qPCR comparing the DNA amounts of the fungal gene *MoPot2* and the rice gene *OsUG*. Data are shown as mean ± SD (Different letter indicate a significant difference *P* < 0.05, n = 9). Experiments were repeated three times with similar results. **E** and** F** Spray and punch inoculation of *WG7* overexpression lines and wild type (WT) plants with *M. oryzae* strain FJ802^*AvrPik-D*^ (with *AvrPik-D*). Scale bars = 0.5 cm. Relative fungal biomass of *M. oryzae* in inoculated leaves, as quantified by qPCR comparing the DNA amounts of the fungal gene *MoPot2* and the rice gene *OsUG*. Data are shown as mean ± SD (Different letter indicate a significant difference *P* < 0.05). **C**, **D** and **G**, **H** Chitin-induced ROS burst in *wg7*, *WG7* overexpression and wild type (WT) plants. **C** and **G** Leaf disks were treated with water or 8 nM chitin, and ROS accumulation was measured by luminol assay. Data are shown as mean ± SD (n ≥ 6). **D** and **H** Calculate the maximum ROS value for each individual leaf disk. Data are shown as mean ± SD (Different letter indicate a significant difference *P* < 0.05, n ≥ 6). **I** Expression of *OsWRKY45* and *OsMT2b* in the leaves of *wg7*, *WG7* overexpression lines and wild type (WT) plants under normal conditions. Data were normalized to the expression level of *OsActin1*. Data are shown as mean ± SEM (The letters a to d indicate a significant difference *P* < 0.05, n = 3)

To investigate the function of WG7 in rice immunity, we generated *wg7* knockout (KO) mutants in the *Pikh*-lacking rice cultivar Nipponbare (NPB) using clustered regularly interspaced short palindromic repeats (CRISPR)/CRISPR-associated nuclease 9 (Cas9)-mediated gene editing. Two KO mutants (*wg7-4#* and *wg7-10#*) in the T3 generation harboring homozygous mutations in *WG7* gene were obtained (Additional file 1: Fig S3A). In a previous study, WG7 has been shown to regulate seed width (Huang et al. 2020), we measured the grain width of the *wg7* mutant, and result showed the grain width were significantly decreased in *wg7* mutants (Additional file 1: Fig S3B, S3C). Next, the *wg7* homozygous mutants were used for blast inoculation assays with *M. oryzae* strain FJ802, which is compatible with all the cultivars tested in this study, transformed with AvrPik-D to generate FJ802^*AvrPik-D*^ strain. Upon conducting a punch inoculation assay, it was observed that the *wg7* mutants developed smaller lesions and lower relative fungal biomass compared with the wild type (Fig. 3A, B). These results indicate that the *WG7* knockout mutants exhibited more resistance to *M. oryzae* than the wild type. Subsequently, the ROS production following the chitin elicitor treatment were assessed. We found that the chitin-induced ROS accumulation was significantly higher in the *wg7* mutants when compared to the wild type (Fig. 3C). Moreover, the maximum ROS value in the *wg7* mutants was also noticeably higher (Fig. 3D).

To further analyze the function of *WG7*, we generated the *WG7*-overexpressing transgenic lines in the NPB background under the control of the maize Ubiquitin promoter. Two *WG7* overexpression (OE) transgenic lines (#4 and #12) were identified by qRT-PCR (Additional file 1: Fig S3D), and T2 plants were used for further analyses. The spray inoculation assays with FJ802^*AvrPik-D*^ strains revealed that the *WG7*-OE lines were more susceptible to the blast pathogen as compared to both the wild type and *wg7* mutants, and higher relative fungal biomass in the *WG7*-OE lines than wild type and *wg7* mutants (Fig. 3E). The punch inoculation assay with FJ802^*AvrPik-D*^ strains showed that the *WG7*-OE mutants developed a larger lesion area and higher relative fungal biomass compared with the wild type (Fig. 3F). Furthermore, the *WG7*-OE plants exhibited lower chitin-induced ROS generation than that in wild type (Fig. 3G), and the maximum ROS value was significantly lower in the *WG7*-OE plants (Fig. 3H). To comprehend the *WG7*-mediated mechanism of rice resistance, we measured the expression of both defense-related genes and ROS scavenger gene regulators in the *WG7* knockout and overexpression lines using qRT-PCR. The analysis showed that, the expression of positive rice resistance regulator, *OsWRKY45* (Shimono et al. 2012; Ueno et al. 2013), was significantly downregulated in OE lines and was upregulated in KO lines; *OsMT2b* is an ROS scavenger (Wong et al. 2004), its expression was lower in the KO lines and was higher in OE lines than in wild type plants (Fig. 3I). Taken together, these results suggest that *WG7* plays a negative role in basal resistance against *M. oryzae* in rice.

### WG7 involved in the regulation of plant hormones SA and JA

**Fig. 4:**
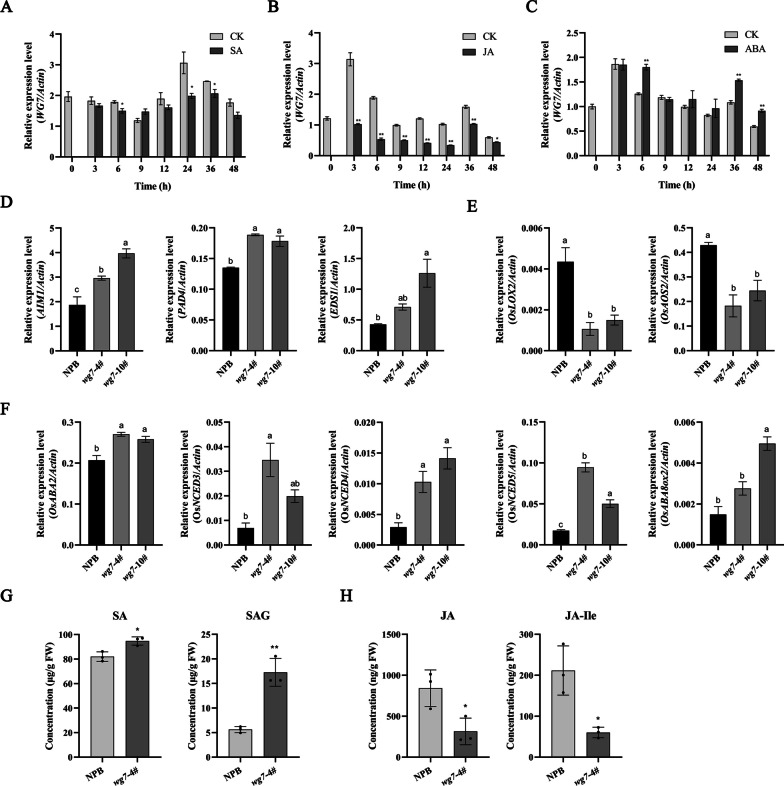
Effect of SA treatment on **A** expression of *WG7*, **B** expression of JA treatment, and **C** expression of ABA treatment. **D** *AIM1*, *PAD4*, and *EDS1* are the SA synthesis-related genes. **E** *OsLOX2* and *OsAOS2* are the JA synthesis-related genes.** F** qRT-PCR gene expression of genes for ABA biosynthesis viz., *OsABA2*, *OsNCED3*, *OsNCED4*, and *OsNCED5*, ABA catabolism (*OsABA8ox2*). Data are from qRT-PCR analysis and show expression levels relative to the controls. *OsActin1* was used as the internal reference gene. qRT analysis data are shown as mean ± SEM (*P < 0.05, **P < 0.01, or the different small letter indicate* P* < 0.05, n = 3). **G**, **H** the content of SA, SAG, JA and JA-Ile in rice plants. FW, Fresh weight. *indicates significant difference between *wg7* plants and the control plants at *P < 0.05, **P < 0.01 (mean ± SD, n = 3)

Plant hormones have crucial roles in the regulation of plant growth, development, and defense (Pieterse et al. 2012). Given that SA, JA, and ABA represent the primary defense signaling molecules, we studied the expression of *WG7* using qRT-PCR following individual treatment with these hormones. Our analysis showed a decrease in *WG7* transcript levels at 6 h, persisting from 24 to 36 h post-SA treatment, compared to the control treatment (Fig. 4A). The levels of *WG7* transcripts began to decrease as early as 3 h and reached their lowest level from 6 to 24 h after JA treatment (Fig. 4B). However, the transcript level of *WG7* significant increase at 6 h, no significant change in 9–24 h, and then continue to increase in 36–48 h after ABA treatment (Fig. 4C). These results suggest that WG7 might be involved in the regulation of plant hormones.

To further confirm the WG7 was involved in regulating the biosynthesis of SA, JA, and ABA, we first verify the expression of marker genes in the pathway of SA, JA, and ABA using qRT-PCR. The result showed transcript levels of the SA synthesis-related genes *AIM1* (ABNORMAL INFLORESCENCE MERISTEM 1), *PAD4* (phytoalexin deficient 4), and *EDS1* (enhanced disease susceptibility 1) (Bo Du, 2009; Xu et al. 2023) were higher in the *wg7* mutants than in wild type plants (Fig. 4D). However, transcript levels of the JA synthesis-related genes *OsLOX2* (lipoxygenase) and *OsAOS2* (allene oxide synthase 2) (Deyun Qiu et al. 2007; Bo Du 2009) were substantially lower in the *wg7* mutants than in wild type plants (Fig. 4E). In addition, WG7 KO mutants showed significantly higher expression levels of gene expression of genes for ABA biosynthesis viz., *OsABA2*, *OsNCED3*, *OsNCED4*, *OsNCED5* and ABA catabolism (*OsABA8ox2*) (Endo et al. 2014; Verma et al. 2019; Zhang et al. 2020) as compared with wild type plants. These results suggest that WG7 might be involved in regulating the biosynthesis of SA, JA, and ABA. To validate the involvement of WG7 in the regulation of plant hormones, we analyzed hormonal profiles in both the wild type and KO mutant. The results indicated a significant increase in SA and SAG (salicylic acid-2-O-β-d-glucose) levels in the KO mutant compared to the wild type (Fig. 4G). Compared to wild type, JA and JA-Ile (*N*-[(−)-jasmonoyl]-(*S*)-isoleucine) levels significantly decreased in the KO mutant (Fig. 4H), and ABA levels was higher in KO mutant than wild type (Additional file 1: Fig S4A). Interestingly, there was a significant decrease in IAA levels in the KO mutant (Additional file 1: Fig S4B), indicating WG7 might regulate plant growth, and we observed visible growth changes of the *wg7* mutant compared to the wild type in 10-day-old rice seedlings (Additional file 1: Fig S4C, D). Previous studies have indicated that the plant height and panicle length of the *wg7* mutant dramatically decrease at the flowering stage, compared with the wild type under the Hwayoung (HY) background (Huang et al. 2020). Taken together, these results indicate that WG7 is involved in the regulation of plant hormones.

### AvrPik-D enhances the transcriptional activity of WG7

**Fig. 5:**
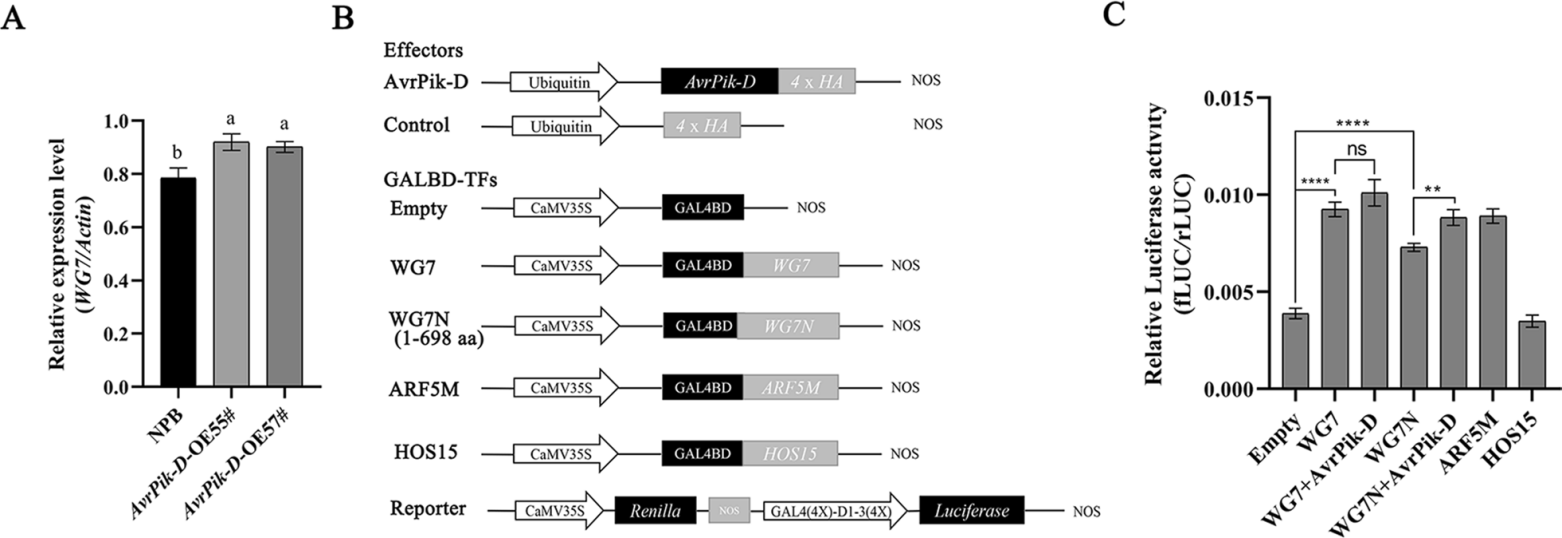
AvrPik-D enhances WG7’s transcriptional activity. **A** qRT-PCR detected the expression of *WG7* in the leaves of *AvrPik-D* overexpressed lines. Data were normalized to the expression level of *OsActin1*. Data are shown as mean ± SEM (Different letter indicate a significant difference, *P* < 0.05, n = 3). **B** Constructs used in the transcriptional activity assays. Coding regions of WG7 and WG7^1−698^ were fused with *GAL4BD* under the control of the CaMV 35S promoter. The *Firefly luciferase* gene driven by four tandem copies of the GAL4 DNA binding site fused immediately upstream of four tandem copies of the constitutive D1-3 element (GAL4(4X)-D1-3(4X)), and the *Renilla luciferase* gene driven by the CaMV 35S promoter was used as the reporter and internal control, respectively. **C** The transcriptional activity assay of WG7 and WG7^1−698^ in rice protoplasts. *HOS15*, a transcription suppressor, and *ARF5M*, a transcription activator, were used as the controls. Asterisks indicate a significant difference between the empty vector with WG7 and WG7^1−698^ (Student’s t-test, **P < 0.01, ****P < 0.0001, n = 3)

To understand the relationship between AvrPik-D and WG7, we tested the expression of *WG7* in *AvrPik-D* overexpressed transgenic rice using qRT-PCR. The result showed that *WG7* transcript levels was significantly increased (Fig. 5A) in the *AvrPik-D* OE lines (Additional file 1: Fig S5) compared to NPB. These results suggest that *AvrPik-D* promote *WG7* at the transcriptional level.

Previous studies have shown that *WG7* is a transcriptional activator involved in regulating gene expression (Huang et al. 2020). To examine whether AvrPik-D affects the transcriptional activity of WG7 *in planta*, we used the GAL4 DNA-binding domain (GAL4BD) and its binding site [GAL4(4X)-D1-3(4X)]-based transient expression system, which was transfected in rice protoplasts (Wang et al. 2016; Wang et al. 2021a, b). The full-length *WG7* and N-terminal fragment (*WG7N*^1−698 aa^) were fused with GAL4BD under the control of the 35S promoter to generate the *GAL4BD*-*WG7* and *GAL4BD*-*WG7N* respectively. The firefly luciferase reporter gene driven by four copies of the GAL4 DNA-binding site [GAL4(4x)-D1-3(4x)-fLUC] was used as a reporter with the renilla luciferase gene under the control of the 35S promoter as internal control (Fig. 5B). ARF5M and HOS15 were used as the controls for transcription activator and repressor, respectively (Guo et al. 2013; Wang et al. 2021a, b). Compared to the Empty control (GAL4BD), the luciferase activity of GAL4BD fused to both full-length WG7 and WG7N were considerably higher, indicating that WG7 is a transcriptional activator and the WG7 N-terminal has transcriptional activity function in rice. The fLUC/rLUC ratio was significantly enhanced when WG7N and AvrPik-D plasmids were co-expressed (Fig. 5C). These results indicated that AvrPik-D enhances the transcriptional activity of WG7 in rice cells.

### WG7 is not essential for Pikh-mediated recognition to AvrPik-D

**Fig. 6:**
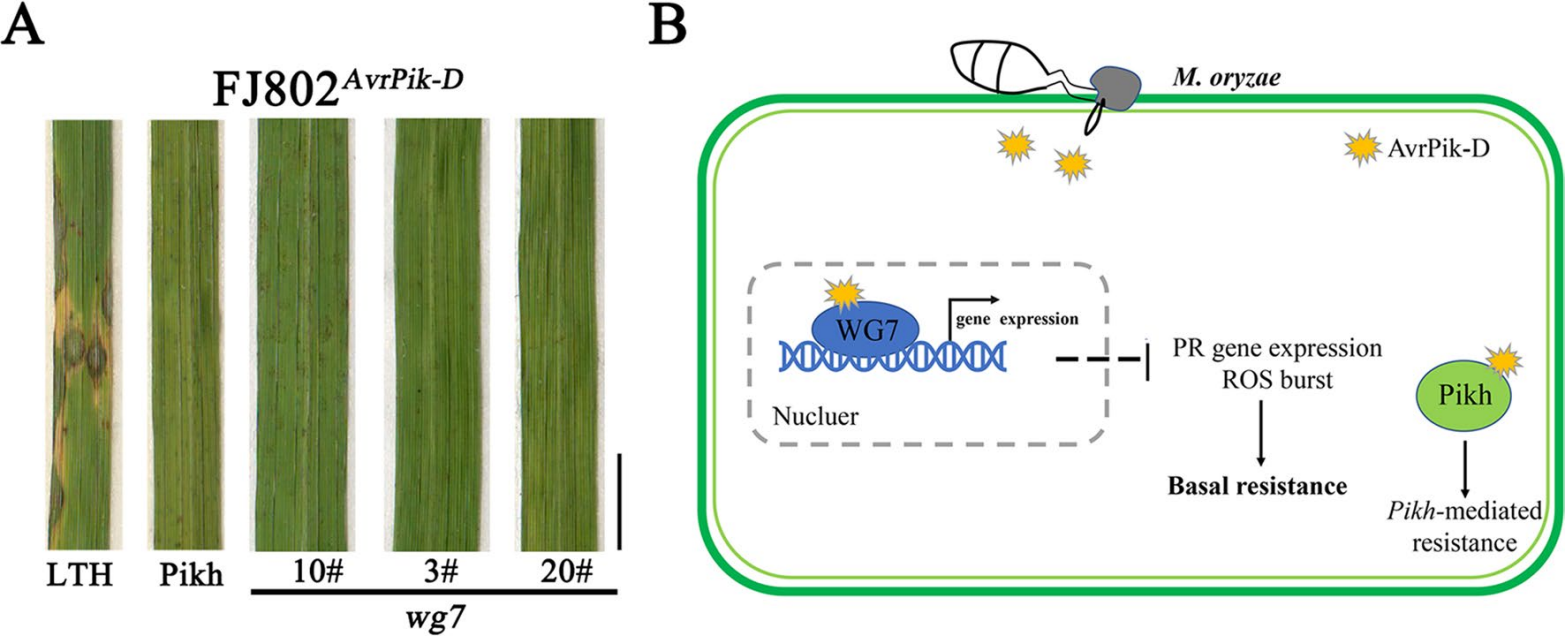
WG7 is not essential for *Pikh*-mediated blast resistance. **A** Spray inoculation of LTH, Pikh and *WG7* mutants in the Pikh background with *M. oryzae* strain FJ802^*AvrPik-D*^ (with *AvrPik-D*). LTH was used as susceptible control. The photographs were taken at 7 days post inoculation. **B** Working model of WG7-mediated basal resistance against *M. oryzae* in rice. During infection, AvrPik-D is secreted into rice cells by *M. oryzae*. AvrPik-D targets the host nucleus to promote transcriptional activity of WG7, Meanwhile, the expression of defense-related genes and the ROS burst are reduced by WG7, which negatively regulates basal resistance against *M. oryzae* in rice

*Pik-h* is the cognate *R* gene of AvrPik-D in rice (Zhai et al. 2014; De la Concepcion et al. 2021). In our study, we found that AvrPik-D and Pikh collectively target WG7 in rice (Fig. 1). To assess whether WG7 contributes to Pikh's recognition of AvrPik-D, we used CRISPR/Cas9-mediated gene editing to generate *wg7* knockout mutants in the Pikh background (containing the *Pikh* gene). We obtained three knockout mutants (*wg7*-3#, -10# and -20#) in the T2 generation harboring homozygous mutations in *WG7* (Additional file 1: Fig S6B). Spray inoculation assays of *wg7* mutants were then performed using *M. oryzae* strain FJ802^*AvrPik−D*^ (Additional file 1: Fig S6A). The spray inoculation assay indicated that both the Pikh and the *wg7* mutants exhibited typical symptoms of resistance to rice blast. In contrast, LTH showed symptoms characteristic of susceptibility as a control (Fig. 6A). This result suggesting that *WG7* is not essential for Pikh-mediated blast resistance in rice.

## Discussion

### The transcription factor WG7 negatively regulates rice immunity to *M. oryzae* through SA signaling pathway

Many CW domain-containing proteins are involved in human diseases (Hong et al. 2017; Wang et al. 2021a, b). CW domain-containing proteins are implicated in a variety of biological processes in plants. For example, *SET DOMAIN GROUP8* (*SDG8*), containing a CW domain, has been shown to regulate flowering time and shoot branching in *Arabidopsis*, and plants with *sdg8* mutations exhibit early flowering and increased shoot branching (Kim et al. 2005; Dong et al. 2008). Berr et al. found that the loss-of-function mutant *sdg8-1* exhibits reduced resistance to the necrotrophic fungal pathogens *Alternaria brassicicola* and *Botrytis cinereal* (Berr et al. 2010). *WG7*, also known as *OsCW-ZF7*, no homology with the *SDG8* by protein sequence alignment, is a rice CW domain-containing protein previously reported to regulate awn development in the cv. Kasalath background (Zhang et al. 2017). However, no information has been reported yet on the role of rice CW domain-containing proteins in plant immunity.

In this study, we show that the *WG7* knockout plants present more resistance than the wild type plants against *M. oryzae* (Fig. 3A, B). In contrast, overexpression of *WG7* increased susceptibility to *M. oryzae* compared with wild type plants (Fig. 3E, F). And the levels of ROS were constitutively up-regulated in the *WG7* knockout plants and down-regulated in the *WG7* overexpression plants (Fig. 3C, G). Lastly, the expression of the defense-related gene, *WRKY45*, was elevated in the *WG7* knockout plants but suppressed in *WG7* overexpression plants (Fig. 3I). By contrast, the ROS scavenger gene, *OsMT2b*, was down-regulated in the *WG7* KO lines and was up-regulated in the *WG7* OE lines (Fig. 3I). Taken together, these results suggest a possible role for WG7 in negatively regulating rice's resistance to *M. oryzae*.

In rice, several plant hormones such as Salicylic acid (SA), Jasmonic acid (JA) and Abscisic acid (ABA) play crucial roles in blast resistance. SA/BTH-induced *OsWRKY45* was regulated by the MAPK-dependent phosphorylation in one of the two branches of the rice SA pathway (Nakayama et al. 2013; Ueno et al. 2013). Activation of ETI and systemic acquired resistance (SAR) often induces the biosynthesis of SA (Meng and Zhang 2013; Hong et al. 2019). In this study, we found that the *WG7* transcript level responds to SA treatment (Fig. 4A). Moreover, the transcript level of Os*WKRY45* was up-regulated in the *WG7* knockout plants and was suppressed in the *WG7* overexpression plants (Fig. 3I). These results suggest that *WG7* might participate in the regulation of the plant hormone SA. To confirm this result, we first analyze the expression of marker genes in the pathway of SA by qRT-PCR, the result showed transcript levels of the SA synthesis-related genes *AIM1*, *PAD4* and *EDS1* were higher in the *wg7* mutants compared to the wild type plants (Fig. 4D). Next, we examined plant hormone levels in both the wild type and KO mutant plants. The results showed that SA and SAG levels were significantly higher in the KO mutants compared to the wild type plants (Fig. 4G). These results suggest that WG7 is involved in SA-mediated resistance against to *M.oryzae*. JA and its derivatives, including its methyl ester (MeJA) and its isoleucine conjugate (JA-Ile), are collectively called jasmonates (JAs) (Ruan et al. 2019). Does et al. found that antagonism between the defense hormones SA and JA plays a central role in the modulation of the plant immune signaling network (Van der Does et al. 2013). Consistent with this, we found JA and JA-Ile levels were significantly decreased in KO mutant (Fig. 4H). Previous studies have shown the plant height of the *wg7* mutant dramatically decreased at the flowering stage compared to the wild type in the 'Hwayoung’ (HY) background (Huang et al. 2020). We also tested the IAA levels and found that there was a significant decrease in the KO mutant than wild type (Additional file 1: Fig S4B) and there were visible growth changes in the KO mutant compared to the wild type in 10-day-old rice seedlings (Additional file 1: Fig S4C, S4D). These results suggest that WG7 is also involved in the regulation of IAA levels, thus affecting the rice growth. Taken together, the above results suggest that WG7 may participate in regulating plant hormones.

### AvrPik-D suppresses rice immunity by targeting and promoting transcriptional activity of WG7

Pathogenic bacteria and fungi secrete a variety of effector proteins to interfere with host defense responses and promote infection (Dodds and Rathjen 2010). Studies of bacteria-plant interactions have shown that bacterial pathogens manipulate host immunity through their effectors (Deslandes and Rivas 2012; Han et al. 2021). For example, *Xanthomonas oryzae* pv. *oryzicola* (*Xoc*) effector AvrRxo1 targets and degrades rice OsPDX1 (pyridoxal phosphate synthase) to inhibits host stomatal immunity (Liu et al. 2022). In the interaction between rice and *M.oryzae*, researchers found *M.oryzae* effector target various rice proteins to suppressed basic resistance. Such as, AvrPiz-t targets the nucleoporin protein APIP12/the K^+^ channel protein OsAKT1 and Avr-Pii targets rice NADP-malic enzymes to suppress basal resistance (Singh et al. 2016; Tang et al. 2017; Shi et al. 2018). AvrPik binds the rice HMA proteins OsHIPP19 and OsHIPP20 to stabilize their proteins to suppresses host defenses (Oikawa et al. 2020; Maidment et al. 2021). However, the target protein of AvrPik-D to promote blast fungal infection remains unclear. In this study, we found the fungal effector AvrPik-D targets the host nucleus and interact with the transcription factor WG7 to suppress rice immunity. In addition, Avr1CO39 and AvrPii interact with WG7 in Y2H assay. These results suggest that Avr1CO39 and AvrPii could potentially manipulate the host defense response by targeting WG7 (Additional file 1: Fig S1D).

Studies report that methylations of histone H3 lysine 9 (H3K9) and H3K27 residues are associated with gene repression, whereas H3K4 and H3K36 methylations are thought to serve as active chromatin marks (Kouzarides 2007; Liu et al. 2010; Zhang et al. 2017). H3K4me3 is highly enriched at the transcription start site (TSS) and can be found at both inactive and active promoters, and may thus play a functional role in the initiation of transcription (Black et al. 2012). In *Arabidopsis*, H3K4 methylation generally binds to the promoters and transcribed regions of genes, regulating gene expression (Zhang et al. 2009). Previous research reported that WG7 directly binds to the cis-element CATTTC motif in the promoter of *OsMADS1*, significantly activating its expression through enhanced histone H3K4me3 enrichment (Huang et al. 2020). Zhang et al. found that the CW domain from WG7 specifically recognizes the trimethylated histone H3 lysine 4 (H3K4me3) and play a crucial role in transcriptional regulation during plant development (Zhang et al. 2017). In our study, we found that the CW domain of WG7 is important for interaction with AvrPik-D (Fig. 1B), and AvrPik-D targets the N-terminal WG7^1−698^ (with CW domain) to promote its transcriptional activity (Fig. 5C). These results suggest that CW domain of WG7 may be important for gene expression regulation and protein interactions. Further studies are needed to determine their biochemical association. In addition, identifying the downstream target gene will assist researchers in determining the function of WG7 in rice immune responses.

Based on these results, we propose a working model for the role of the effector AvrPik-D in interfering the host innate immunity. During rice cell invasion, *M. oryzae* secretes AvrPik-D into rice cells, targeting the host nucleus to promote gene expression and transcriptional activity of WG7, subsequently reducing the ROS burst and SA level, increasing JA and IAA levels, to damage rice immunity. Furthermore, WG7 is not essential for Pikh-mediated blast resistance (Fig. 6B).

## Conclusions

In summary, WG7, a nuclear-localized transcription factor, negatively regulates the plant immunity by reducing the ROS burst and increasing susceptibility to the pathogen. AvrPik-D targets the host nucleus and interacts with WG7 to promote its transcription activity to suppress the PTI.

## Materials and methods

### Plant materials and growth conditions

Rice seeds were soaked in water at room temperature for 2 days and then germinated, in an incubator at 37 °C. After germination, the seeds were planted in small pots and were grown in a growth chamber under conditions of 12-h light, 28 °C, 70% relative humidity followed by 12-h darkness, 26 °C. *N.benthamiana* was grown in a growth chamber at 24 °C with 16 h of light and 8 h of darkness.

### Yeast two-hybrid screening and Interaction verification

The full-length coding region of AvrPik-D (lacking the N-terminal signal peptide, ΔSP) was cloned into the pGBKT7 vector that which contain a binding domain (BD), then BD-AvrPik-D plasmid was transformed into AH109 competent cells and plated onto SD-Trp, SD-Leu-Trp-His or SD-Leu-Trp-His-Ade plates to test self-activation following the manufacturer’s instructions. Next, AH109 strains containing bait plasmid was used for making competent cells, the cDNA library was transformed into competent cells and plated onto SD-Leu-Trp-His-Ade plus X-α-gal plates to select positive clones by incubation at 30 °C for 4–6 d. After obtaining potential targets of AvrPik-D, the full-length CDS of the target genes were cloned into the pGADT7 vector. Subsequently, each potential target was individually tested to verify interaction with AvrPik-D using a Y2H assay.

### Pull down assay

The GST pull down assay was performed as described by Liu with some modifications (Liu et al. 2021). Briefly, the open reading frame of *AvrPik-D* (ΔSP) was inserted into pMAL-c2X (containing N-MBP tag) vector and the *WG7*^1−698^ fragment was cloned into pGEX-4T-1 vector (containing N-GST tag) using the ClonExpress II One Step Cloning Kit (Vazyme, Nanjing, China). The constructs were separately transformed into *E. coli* BL21 (DE3). The *E. coli* containing expression plasmid for protein were incubated at 37 °C and 200 rpm until OD_600_ was reached 0.4 to 0.6, then there was further incubation at 16 °C for 16 h by the addition of 0.4 mM Isopropyl-beta-D-thiogalactopyranoside (IPTG). Cells were harvested and resuspended in GST binding buffer (140 mM NaCl, 2.7 mM KCl, 10 mM Na_2_HPO_4_, 1.8 mM KH_2_PO_4_, pH 7.4, 1 mM PMSF, 5 mM DTT and 1 × protease inhibitor cocktail) for GST-WG7^1−698^ protein isolation, and MBP binding buffer (20 mM Tris–HCl, 200 mM NaCl, 1 mM EDTA, pH 7.4, 1 mM PMSF, 5 mM DTT and 1 × protease inhibitor cocktail) for MBP or MBP-AvrPik-D isolation. The suspended cells were performed ultrasonic lysis and then the mixture of broken cells was centrifuged at 12,000 rpm at 4 °C for 20 min and the supernatant was collected for further analysis. GST-WG7^1−698^ was incubated with GST beads at 4 °C for 4 h and then the GST beads were washed 3 times. The aliquot of beads was added MBP or MBP-AvrPik-D, then incubate for another 4 h at 4 °C. Input and pull-down proteins were electrophoresed in sodium dodecyl sulphate polyacrylamide gel (SDS-PAGE) followed by Western blot analysis with corresponding antibodies.

### Co-IP assay

Both the CDS of *WG7N*^*1−698*^ (with GFP tag) and *AvrPik-D* (ΔSP, with mCherry tags) were cloned into *pCXSN* vector, driven by the 35 S promoter (Chen et al. 2009). The WG7N-GFP and AvrPik-D-mCherry vector was individually transformed into Agrobacterium tumefaciens strain GV3101, and then various combinations were infiltrated into *N.benthamiana* leaves. The infiltrated leaves were harvested after 48–72 h and then ground to powder in liquid nitrogen. The powder was resuspended in lysis buffer (10 mM Tris–HCL, pH7.5, 0.5 mM EDTA, 150 mM NaCl, 0.5% NP40, 1 mM PMSF, and 1 × protease inhibitor cocktail). The GFP beads were added to the protein lysates and gently rotated at 4 °C for 2–4 h. The beads were then washed for 3 to 5 times. The beads mixtures were collected for Western blot. WG7N-GFP and AvrPik-D-mCherry fusion proteins were detected by anti-GFP (Abmart) and anti-mCherry (Abmart) antibodies, respectively.

### Subcellular localization

Subcellular localization analysis of various fusion proteins was performed using a transient expression system. The full-length CDS of WG7 was inserted into pHF225 vector with C-terminal RFP and AvrPik-D (ΔSP) inserted into pRTVnGFP vector with N-terminal GFP, driven by the *maize* ubiquitin promoter. Rice protoplasts were prepared and plasmid transformation was performed according to the methods of Wang et.al (Guo et al. 2013; Wang et al. 2016). Fluorescent signals were observed using confocal laser scanning microscopy (NIKON A1, Japan) with excitation at 488 nm and 561 nm, respectively. For subcellular localization in *N. benthamiana*, the corresponding plasmids were transformed into *Agrobacterium tumefaciens* strain GV3101 and transiently infiltrated into the leaves of 4-week-old *N.benthamiana*. The inoculated leaves were incubated for 48–72 h at room temperature before observation.

### RNA isolation and qRT-PCR

Total RNA was extracted from various rice leaves tissues using Eastep® Super Total RNA Extraction Kit (Cat NO.LS1040, Shanghai Promega) according to the manufacturer’s instructions. 1 μg total RNA was reverse transcribed into first-strand cDNA using *Evo M-MLV* Mix Kit with gDNA Clean for qPCR (Accurate Biology, AG11728, China) according to the manufacturer’s protocol. Transcript levels were quantified by qRT-PCR using SYBR Green Premix *Pro Tag* HS qPCR Kit (Accurate Biology, AG11701, China). The relative quantification of the candidate gene's expression level was normalized to that of *OsACTIN1* or *OsUBQ5* using the 2^−ΔCt^ method (Han et al. 2021). The specific sequences of the primers used are provided in Additional file 2: Table S1.

### Construction of transgenic rice plants

Two specific targets within the *WG7* gene were designed using the *CRISPR-GE* toolkit (Xie et al. 2017) for CRISPR/Cas9-based genome editing system to generate the *WG7* gene knockout transgenic plants. The gene editing result was further checked by sequencing of the amplified targeted sequence and homozygotes were selected for phenotypic analysis. To construct *WG7* overexpression vector, the full-length CDS of *WG7* was amplified from rice cDNA of NPB with the primers OE-WG7-F and OE-WG7-R (Additional file 2: Table S1) and then cloned into a *BamHI*-linearized *pCXUN* plasmid (Chen et al. 2009). These plasmids were then transformed into the *Agrobacterium tumefaciens* strain EHA105 and subsequently were used as *Agrobacterium*-mediated genetic transformation. Transgenic rice plants were generated in the Nipponbare background and rice transformations were performed by Wuhan BioRun Biosciences Co., Ltd. (Wuhan, China).

### Transformation of *M. oryzae* isolate and Pathogen Inoculation assay

To construct the *pKNT*-*AvrPik-D* expression vector, *AvrPik-D* was amplified by PCR from genomic DNA of *M. oryzae* FJ81278 under the native promotor using the primers *pKNT*-*AvrPik-D*-CF and *pKNT*-*AvrPik-D*-CR (Additional file 2: Table S1), then cloned into the *KpnI* and *HindIII*-linearized *pKNT* plasmid. The constructed vector was transformed into *M. oryzae* FJ802 protoplasts following a previously described method (Sugihara et al. 2023). The *Magnaporthe oryzae* isolates FJ802 and FJ802^*AvrPik-D*^ were cultured on complete medium for 1 week in the dark at 28 °C, then cut the mycelium into small pieces and were moved to rice bran culture-medium for sporulation. Spore concentration was adjusted using a hemacytometer before spores were used by spraying or punch inoculation (Park et al. 2012).

The punch inoculation according to the method of Park et al. (2012) with a slight modification. A 10-μL volume of a spore suspension (5 × 10^5^ spores/mL^−1^) was applied to slightly punctured sites of leaves on 6 to 8 weeks old plants. Lesion diameters were measured at 10 days using ImageJ software. For spraying inoculation assays, the concentration of the conidial suspension was adjusted to 2 × 10^5^ per mL with 0.02% Tween-20. Four-week-old rice seedlings were used for spray inoculation and the symptoms were assessed 7 days post-inoculation. The fungal biomass in infected rice leaf tissue was quantified used a previously described method (Park et al. 2012). In brief, a small piece of infected rice tissue (about 4 × 1 cm) was cut for DNA extraction using the standard CTAB extraction method. DNA-based qPCR was performed and relative fungal growth was calculated using the threshold cycle value (C_T_) of *M. oryzae Pot2* DNA against the C_T_ of rice genomic *ubiquitin* DNA. Relative fungal growth was then calculated represented by the Eq. 2^CT(*Os-UBQ*)−CT(*Mo-Pot2*)^.

### Measurement of ROS

ROS measurement using the luminol chemiluminescence assay was performed as previously described (Park et al. 2012) with some modifications. Briefly, leaf disks from 6-week-old plants were cut by a 6 mm hole punch and pre-incubated overnight in sterile distilled water. The leaf disks were transferred to a 96-well microplate, 8 nM chitin or water was added to the reaction buffer containing 20 mM of luminol (Wako) and 5 mg/mL of horseradish peroxidase (Sigma). Luminescence was monitored immediately after the treatment and was continuously measured at 1-min intervals for 30 min using a Varioskan Flash multireader (Thermo Fisher Scientific).

### Transcriptional activity analysis

The WG7 and WG7N^1−698^ transcriptional activity assay in rice protoplasts were performed as described previously with some modifications (Huang et al. 2020; Wang et al. 2021a, b). In brief, the four GAL4 DNA binding sites with four tandem copies of the D1-3 element (GAL4(4x)-D1-3(4x)) were amplified by PCR and then cloned into the pGreenII 0800-fLUC vector at the *KpnI* and *SpeI* restriction sites to generate the pGreenII 0800-GAL4(4x)-D1-3(4x)-fLUC construct, which includes the Renilla luciferase (*rLUC*) gene driven by 35S promoter and served as the internal control. The *WG7* full length and *WG7N*^1−698^ fragments were fused with GAL4BD under the control of the 35S promoter to form the GAL4BD-*WG7* or GAL4BD-*WG7N*^1−698^ constructs. *Avrpik-D* was cloned into the pRGV-HA vector under the control of the *maize* ubiquitin promoter and used as effectors. Rice protoplasts were isolated from the yellow flower seedling of approximately 10 to 14-days wild type (NPB) plants and polyethylene glycol (PEG)-mediated transformation was used for transfection as described previously (Guo et al. 2013). fLUC activity and rLUS values were measured by a Varioskan Flash Luminometer (Thermo Fisher Scientific) using Dual-Luciferase® Reporter Assay System (E1910, Promega) according to the manufacturer’s instructions. Relative ratios of fLUC/rLUC were used to evaluate the transcriptional activity of WG7 and WG7N^1−698^.

### SA, JA and ABA treatment

To determine the expression level of *WG7* that was induced by SA (Salicylic acid), JA (Jasmonate acid) and ABA (Abscisic acid), the 2-week-old rice seedlings were separately sprayed onto leaves (1 mL/plant) with 1 mM SA (Gao et al. 2017), 500 µΜ Me-JA (Wu et al. 2019) and 50 µM ABA (Zhang et al. 2021) at different times according to previous studies and then kept in an incubator at 28 °C with high humidity. For mock treatments, only the solvents containing 0.5% absolute ethanol and 0.01% Tween 20 were sprayed.

### Phytohormones measurement

Total plant hormones were extracted from the second leaves of 50-day-old plants. Plant hormones were measured as previously described with some modifications (Li et al. 2016; Hui et al. 2018). In brief, rice leaves were ground to a fine powder with liquid nitrogen. Weigh an appropriate amount of lyophilized sample in a 2 mL brown centrifuge tube, add 1 mL of methanol and mixed internal standard stock solution accurately, sonicated for 10 min, transferred to a metal bath and shaken for 4 h, then centrifuged at 12,000 rpm for 10 min at 4 °C, and the entire supernatant was removed after centrifugation. The extracts were centrifuged through a 0.22 μm filter membrane and 5 μL of each extracted solution were injected vial an Acquity UPLC® CSH C18 (1.7 μm, 2.1 × 150 mm, Waters) column for LC–MS/MS (AB Sciex, USA) analysis. The negative ion mode was used to detect the SA, SAG, JA, JA-Ile and ABA, and positive ion mode was used to detect the IAA. The temperature of the column was set at 40 °C. Eluents was consisted of 0.05% formic acid with 2 mM ammonium formate water (A) and 0.05% formic acid methanol (B). The flow rate was set at 0.25 mL/min. An elution gradient was performed as follows: 0–2 min, 10% B; 2–4 min, 10–30% B; 4–19 min, 30–95% B; 19–19.10 min, 95–10% B; 19.10–22 min, 10% B. Phytohormone analysis was performed by the PANOMIX Biomedical Tech Co.,Ltd (Suzhou, China). All experiments were performed with three biological repeats, each biological replicate contained 6 different plants.

### Statistical analysis

Statistical analysis of the experimental data was determined by t-test or one-way ANOVA. Multiple groups of data were compared using one-way ANOVA followed by Tukey’s test with *P* < 0.05 being considered significant using the IBM SPSS Statistics 25.0 software. Two groups of data were compared by two-tailed Student’s t test (*P < 0.05; **P < 0.01; ns, with no significant difference).

### Accession numbers

The sequence data can be found in Rice Genome Annotation Project (http://rice.uga.edu/ index.shtml). *WG7* (LOC_Os07g47360), *OsActin1* (LOC_Os03g50885), *OsUBQ5* (LOC_Os01g22490), *OsWRKY45* (LOC_Os05g25770), *OsMT2b* (LOC_Os05g02070*), AIM1* (LOC_Os02g17390), *PAD4* (LOC_Os11g09010), *EDS1* (LOC_Os09g22450),* OsLOX2* (LOC_Os03g52860), *OsAOS2* (LOC_Os03g12500),* OsABA2* (LOC_Os03g59610), *OsNCED3* (LOC_Os07g05940),* OsNCED4* (LOC_Os07g05940),* OsNCED5* (LOC_Os12g42280), *OsABA8ox2* (LOC_Os08g36860).

### Supplementary Information

**Additional file 1**. Fig. S1 to s6.

**Additional file 2. Table S1.** Primers used in this study.

## Data Availability

All data generated or analysed during this study are included in this published article and its supplementary information files.

## Abbreviations

PTI: Pathogen-associated molecular patterns (PAMPs)-triggered immunity
ETI: Effector-triggered immunity (ETI)
Y2H: Yeast two-hybrid
*WG7*: *Grain Width 7*
*GFP*: Green fluorescent protein
*RFP*: Red fluorescent protein
*H2B*: Histone H2B
ROS: Reactive oxygen species
SA: Salicylic acid
JA: Jasmonic acid
ABA: Abscisic acid
IAA: Auxin
