# Supplementary material for: Magnaporthe oryzae effector AvrPik-D targets a transcription factor WG7 to suppress rice immunity
Source: Rice (N Y). 2024 Feb 13;17:14. doi: 10.1186/s12284-024-00693-0 (PMC10864242; doi:10.1186/s12284-024-00693-0)
Supplement: Supplementary file 1 — Additional file 1. Fig. S1 to s6. [file 12284_2024_693_MOESM1_ESM.docx]

**Supplementary Information**

**Additional file 1:** **Fig S1**


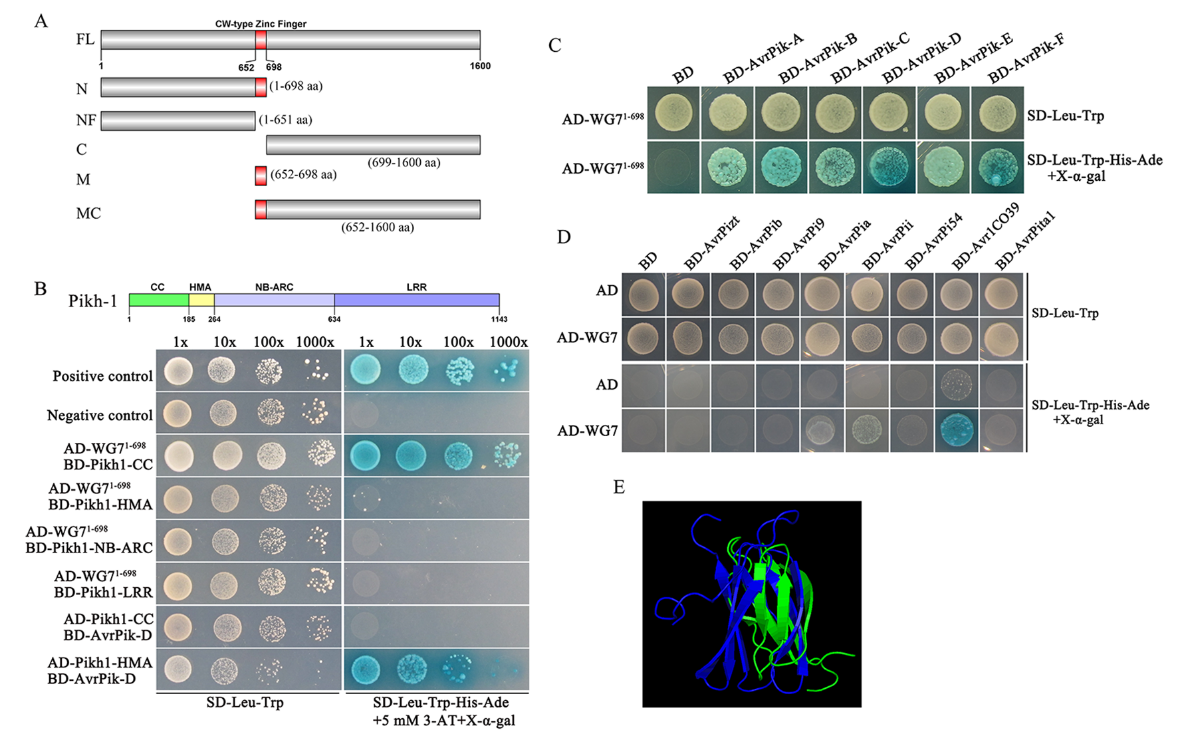


**Fig S1** WG7 is predicted to be a CW-type Zinc Finger protein and interacts with AvrPik alleles.

**A** Schematic diagram of WG7 protein domain truncation. We used NCBI CD-Search website (https://www.ncbi.nlm.nih.gov/Structure/cdd/wrpsb.cgi) to predict CW domain sequences in WG7. **B** Schematic diagram of Pikh1 protein domain and interaction with WG7^1-698^ and AvrPik-D. **C** Detected of protein-protein interactions between BD:AvrPik (ΔSP) alleles and AD:WG7^1-698^ via yeast-two-hybrid (Y2H) assay. Yeast cells harboring the indicated bait and prey plasmids were spotted onto selective media SD-Leu-Trp and SD-Leu-Trp-His-Ade with X-α-gal. A-F indicate AvrPik alleles. SD (-Leu-Trp) indicates selective medium lacking leucine and tryptophan. SD (-Leu-Trp-His-Ade) indicates selective medium lacking leucine, tryptophan, adenine and histidine. **D** Y2H assay to detect the interaction among WG7^1-1600^ with different AVR proteins. **E** Cartoon representations of AVR-Pik (blue, UniProt ID: AF-A0A1S6R4Z6-F1, using the region contain 34-113 aa) and Avr1CO39 (green, PDB ID:5ZNG chain B, using the region contain 22-83 aa) highlight the similar β-sandwich structure of both proteins. The Fig was generated using PyMOL (http://www.pymol.org/).

**Additional file 2: Fig S2**


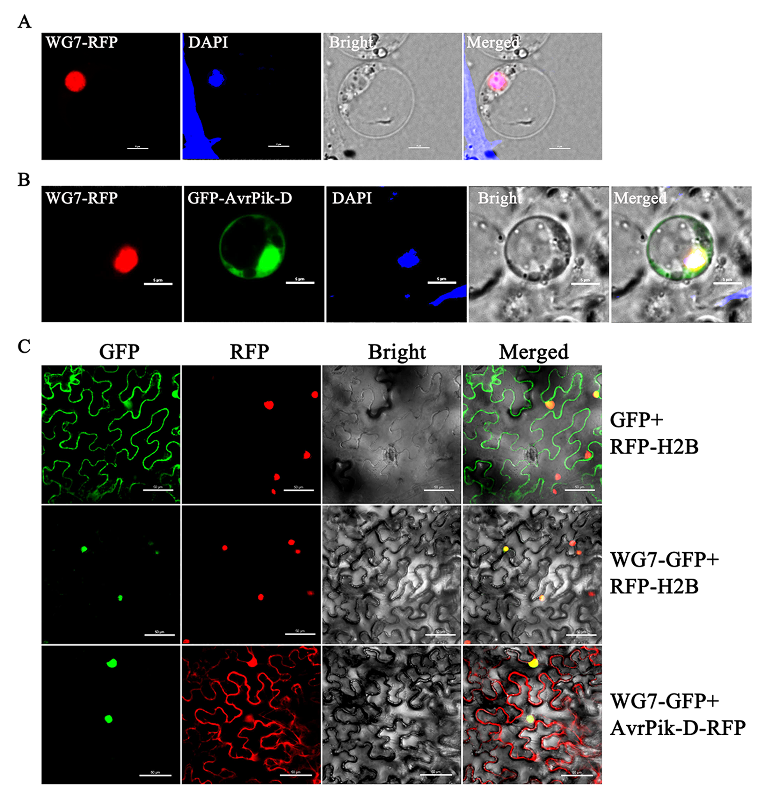


**Fig S2** AvrPik-D and WG7 co-localize to plant nucleus.

**A** WG7:RFP was expressed in rice protoplasts. RFP fluorescent signals and DAPI co-localized in the nucleus. Scale bars = 5 μm. **B** GFP:AvrPik-D and WG7:RFP were co-expressed with DAPI in rice protoplasts. GFP and RFP fluorescent signals overlapped in the nucleus. Scale bars = 5 μm. **C** WG7-GFP and RFP-H2B were transiently co-expressed in *N. benthamiana* leaf both under the control of the CaMV 35S promoter. And then WG7-GFP and AvrPik-D-RFP driven by the CaMV 35S promoter were transiently co-expressed in *N. benthamiana* leaf. GFP empty and RFP-H2B were used as control. Fluorescence was visualized using confocal microscopy. GFP and RFP fluorescent signals overlapped in the nucleus. Scale bars = 50 μm.

**Additional file 3: Fig S3**


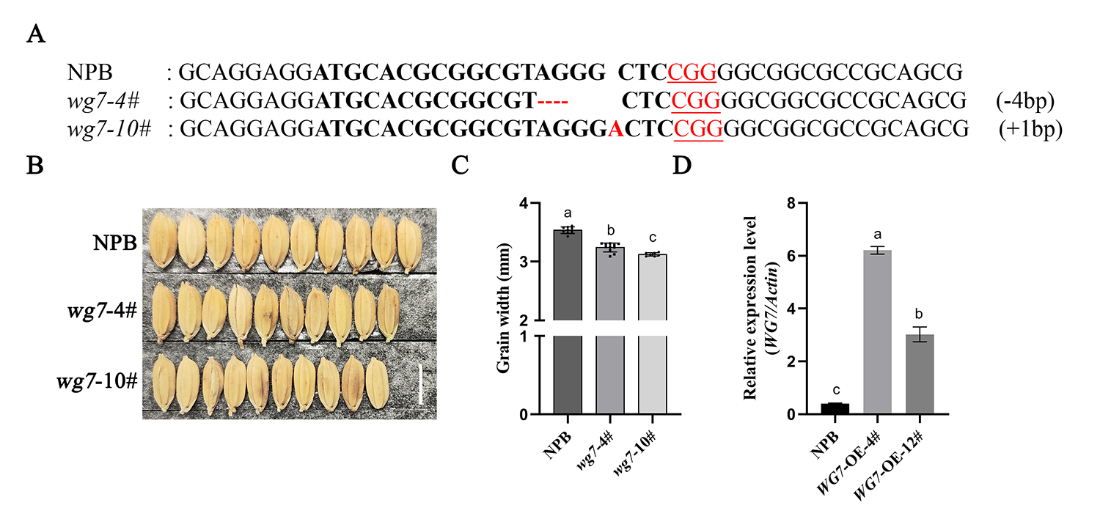


**Fig S3** *WG7* knockout mutant edit types and overexpression detection.

**A** Mutation sites of homozygous *WG7* mutants generated via CRISPR/Cas9 gene editing in NPB. **B** and **C** Grain width of two independent *WG7* knockout plants (T3) and the NPB. Scale bar=0.5 cm. Data are shown as mean ± SD (The letter a-c indicates a significant difference P < 0.05, n≥7). **D** qRT-PCR analysis the gene expression level of *WG7* in *P_ubi_*::*WG7* transgenic plants. Data were normalized to the expression level of *OsActin1*. Data are shown as mean ± SEM (The letter a-c indicate a significant difference P < 0.05, n = 3).

**Additional file 4: Fig S4**


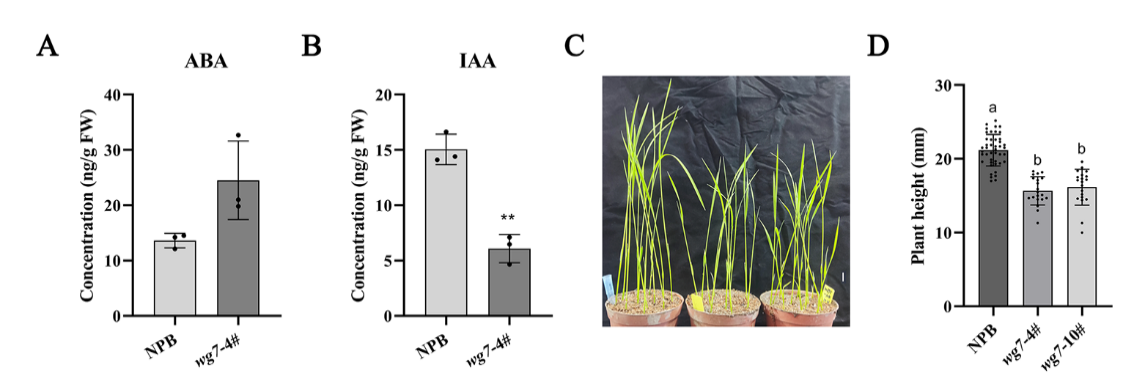


**Fig S4** WG7 involved in plant hormones IAA.

**A** and **B** The content of ABA, IAA in rice plants. FW, Fresh weight. *indicates significant difference between *wg7* plants and the control plants at *P < 0.05, **P < 0.01 (mean ± SD, n = 3).

**C** and **D** Plant height of the *WG7* mutant compared to the control. The images were photographed in 10-day-old seedling. Scale bar =1 cm. The black dots indicate each different plant. Data are shown as mean ± standard error (SD) (The letter a-b indicate a significant difference P < 0.05).

**Additional file 5: Fig S5**


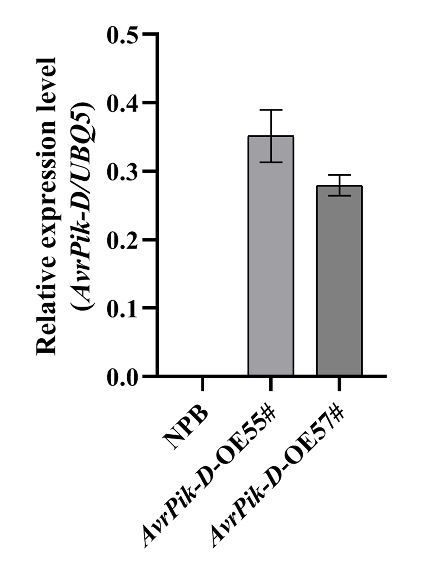


**Fig S5** Expression of *AvrPik-D* in overexpression lines.

qRT-PCR analysis the gene expression level of *AvrPik-D* in *P_ubi_*::*AvrPik-D* transgenic plants. Data were normalized to the expression level of *OsUBQ5*. Data are shown as mean ± SEM (n = 3).

**Additional file 6:** **Fig S6**

**
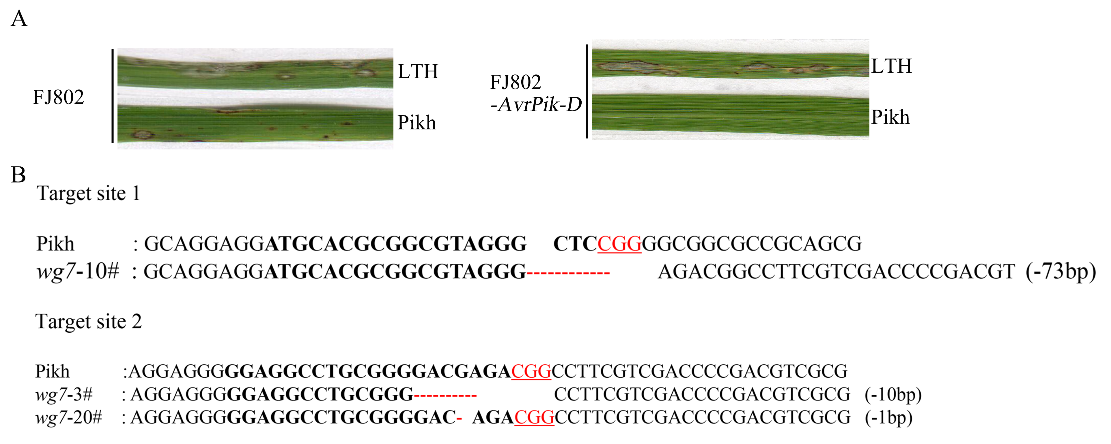
**

**Fig S6** Pathogenicity of Pikh rice against tested strains FJ802 and FJ802*^AvrPik-D^*.

**A** Spray inoculation of LTH and Pikh plants with *M. oryzae* strain FJ802 or FJ802*^AvrPik-D^* (with *AvrPik-D*). LTH was used as susceptible control. The images were photographed at 7 days post inoculation (dpi). **B** Mutation sites of homozygous *WG7* mutants generated via CRISPR/Cas9 gene editing in *Pikh* background.
