# Supplementary material for: Magnaporthe oryzae effector AvrPik-D targets a transcription factor WG7 to suppress rice immunity
Source: Rice (N Y). 2024 Feb 13;17:14. doi: 10.1186/s12284-024-00693-0 (PMC10864242; doi:10.1186/s12284-024-00693-0)
Supplement: Supplementary file 2 — Additional file 2. Table S1. Primers used in this study. [file 12284_2024_693_MOESM2_ESM.docx]

**Supplementary Information**

**Additional file 7: Table S1**

**: Table S1.** Primers used in this study.

| Primer | Sequence (5’-3’) | Described |
| --- | --- | --- |
| BD-*AvrPik-D*-F | tcagaggaggacctgcatatgATGGAAACGGGCAACAAATATATA | Construct vector for Y2H assay |
| BD-*AvrPik-D*-R | gcaggtcgacggatccccgggTTAAAAGCCGGGCCTTTTTT |  |
| AD-*WG7*_1-1600_-F | gtaccagattacgctcatatgATGCTGTCGGTGAGGAGGC |  |
| AD-*WG7*_1-1600_-R | cagctcgagctcgatggatccTCAGCAGCTGATAGACTCCATTGA |  |
| AD-*WG7*_1-698_-R | ccgtatcgatgcccacccgggTCACTCATCTTCACTAACTTCACATCTATT |  |
| AD-*WG7*_1-651_-R | ccgtatcgatgcccacccgggTCATTCCTCTATCACGACGGGGGCAG |  |
| AD-*WG7*_699-1600_-F | gtaccagattacgctcatatgATGACTACCAATGCTCTGAATGCACTATAT |  |
| AD-*WG7*_652-1600_-F | gtaccagattacgctcatatgATGCATTGGGTATGCTGTGACATATGC |  |
| BD-*Pikh1*-F | tcagaggaggacctgcatatgATGGAGGCGGCTGCCATG |  |
| BD-*Pikh1*-R | gcaggtcgacggatccccgggCTAGCTAGTAGTTTCTGTTTGAATTTCAAT |  |
| BD-*Pikh2*-F | tcagaggaggacctgcatatgATGGAGTTGGTGGTAGGTGCTT |  |
| BD-*Pikh2*-R | gcaggtcgacggatccccgggTCATGCAGTGACGATGCCAT |  |
| GST-*WG7*^1-698^-F | gatctggttccgcgtggatccATGCTGTCGGTGAGGAGGC | For Pull-down assay |
| GST-*WG7*^1-698^-R | ctcgagtcgacccgggaattcTCACTCATCTTCACTAACTTCACATCTATT |  |
| MBP-*AvrPik-D*-F | gagggaaggatttcagaattcATGGAAACGGGCAACAAATATATA |  |
| MBP-*AvrPik-D*-R | tgcctgcaggtcgactctagaTTAAAAGCCGGGCCTTTTTT |  |
| GFP-*AvrPik-D*-F | aagggatccccgggtgagctcATGGAAACGGGCAACAAATATATA | For transient expression of rice protoplasts assay |
| GFP-*AvrPik-D*-R | agcggccgcactagtaagcttTTAAAAGCCGGGCCTTTTTT |  |
| pHF225-*WG7*-RFP-F | agatccagtgggatccccgggtATGCTGTCGGTGAGGAGGC |  |
| pHF225-*WG7*-RFP-R | agcggccgcactagtaagcttGCAGCTGATAGACTCCATTGAAAG |  |
| SN-*WG7*-GFP-1F | aacgatactcgagggggatccATGCTGTCGGTGAGGAGGC | For transient expression of *N.benthamiana* assay |
| SN-*WG7*-GFP-1R | cGCAGCTGATAGACTCCATTGAAAG |  |
| SN-*WG7*-GFP-2F | aatggagtctatcagctgcGGATCCCCGGGTGGTCAG |  |
| SN-*WG7*-GFP-2R | gggaaattcgctagtggatccTCAAAGATCTACCATGTACAGCTCG |  |
| *AvrPik-D*-RFP-F | aacgatactcgagggggatccATGGAAACGGGCAACAAATATATA |  |
| *AvrPik-D*-RFP-R | gggaaattcgctagtggatccCTACTTGTACAGCTCGTCCATGCC |  |
| *WG7*_gRT1 | TGCACGCGGCGTAGGGCTCgttttagagctagaaat | For CRISPR/Cas9 gene editing assay |
| *WG7*_OsU3T1 | GAGCCCTACGCCGCGTGCATgccacggatcatctgc |  |
| *WG7*_gRT2 | GAGGCCTGCGGGGACGAGAgttttagagctagaaat |  |
| *WG7*_OsU6aT2 | TCTCGTCCCCGCAGGCCTCCggcagccaagccagca |  |
| *pKNT*-*AvrPikD*-CF | agggaacaaaagctgggtaccTGGGAGCGCGGATAGGCA | For construct the *pKNT*-*AvrPikD* expression vector |
| *pKNT*-*AvrPikD*-CR | gacctgcaggcatgcaagcttGCACGCCACTCCGTAACACC |  |
| OE-*WG7*-F | cttctgcagcccgggggatccATGCTGTCGGTGAGGAGGC | For generate overexpression rice |
| OE-*WG7*-R | cgatcggggaaattcggatccTCACTTGTCATCGTCATCCTTGTAATCGATATCATGATCTTTATAATCACCGTCATGGTCTTTGTAGTCCATGCAGCTGATAGACTCCATTGAAAG |  |
| *OsWG7*-QF | TCTCTTCATCACCACCTA | For qRT-PCR assay |
| *OsWG7*-QR | TGCCGAACATTATCTACTAT |  |
| *OsWRKY45*-QF | CGGGTAAAACGATCGAAAGA |  |
| *OsWRKY45*-QR | TTTCGAAAGCGGAAGAACAG |  |
| *OsM2Tb*-QF | CAGCTTATATGTAGGCAGGC |  |
| *OsMT2b*-QR | GGGATGAAAGCAGAGGTAGA |  |
| *AIM1-QF* | CACTATTGGAGCACCTTAT |  |
| *AIM1-QR* | CTCAATGGTAGGCTTCTT |  |
| *PAD4-QF* | AGGGGTTCTTGAGGCTGTGC |  |
| *PAD4-QR* | GCTGAGCTTGACGATGATGTG |  |
| *EDS1-QF* | CATTCCAAGAACGAGGACACTG |  |
| *EDS1-QR* | CAAGACTCAAGGCTAGAACCGA |  |
| *OsLOX2-QF* | GTGGGAGGTGGAGAAGATGG |  |
| *OsLOX2-QR* | CAGGAGTTGGCGACGAAGA |  |
| *OsAOS2-QF* | AAGCTGCTGCAATACGTGTACTGG |  |
| *OsAOS2-QF* | CGACGAGCAACAGCCTTCCG |  |
| *OsABA2-QF* | GACCTGACGAGACGATGTCC |  |
| *OsABA2-QR* | GCAACCTTGCTTTCCAACC |  |
| *OsNCED3-QF* | *CCCTCCCAAACCATCCAAACCGA* |  |
| *OsNCED3-QR* | *GTGAGCATATCCTGGCGTCGTGA* |  |
| *OsNCED4-QF* | TCCATCTCCTTCTCCCTCCTCCCA |  |
| *OsNCED4-QR* | CCTCGCACCCTGCTTGATCTTGCC |  |
| *OsNCED5-QF* | ACATCCGAGCTCCTCGTCGTGAA |  |
| *OsNCED5-QR* | TTGGAAGGTGTTTTGGAATGAACCA |  |
| *OsABA8ox2-QF* | TTCTTGCTCTTCTTTGTCT |  |
| *OsABA8ox2-QR* | AACATCTCTCCTCCTCTG |  |
| *OsActin1*-QF | TGTATGCCAGTGGTCGTACCA |  |
| *OsActin1*-QR | CCAGCAAGGTCGAGACGAA |  |
| *OsUBQ5*-QF | AACCAGCTGAGGCCCAAGA |  |
| *OsUBQ5*-QR | ACGATTGATTTAACCAGTCCATGA |  |
| *AvrPik-D*-QF | AACGGGCAACAAATATATAG |  |
| *AvrPik-D*-QR | TTTCCAAGAGCTGTAACA |  |
| *OsUG*-F | TTCTGGTCCTTCCACTTTCAG | qRT-PCR of rice  genomic ubiquitin |
| *OsUG*-R | ACGATTGATTTAACCAGTCCATGA |  |
| *MoPot2*-F | ACGACCCGTCTTTACTTATTTGG | qRT-PCR of  *M.oryzae* retrotransposon  Pot2 |
| *MoPot2*-R | AAGTAGCGTTGGTTTTGTTGGAT |  |
